# Relationships between temperaments, occupational stress, and insomnia among Japanese workers

**DOI:** 10.1371/journal.pone.0175346

**Published:** 2017-04-13

**Authors:** Yasuhiko Deguchi, Shinichi Iwasaki, Hideyuki Ishimoto, Koichiro Ogawa, Yuichi Fukuda, Tomoko Nitta, Tomoe Mitake, Yukako Nogi, Koki Inoue

**Affiliations:** Department of Neuropsychiatry, Osaka City University, Graduate School of Medicine, Osaka, Japan; University of Illinois at Chicago, UNITED STATES

## Abstract

Insomnia among workers reduces the quality of life, contributes toward the economic burden of healthcare costs and losses in work performance. The relationship between occupational stress and insomnia has been reported in previous studies, but there has been little attention to temperament in occupational safety and health research. The aim of this study was to clarify the relationships between temperament, occupational stress, and insomnia. The subjects were 133 Japanese daytime local government employees. Temperament was assessed using the Temperament Evaluation of Memphis, Pisa, Paris, and San Diego-Auto questionnaire (TEMPS-A). Occupational stress was assessed using the Generic Job Stress Questionnaire (GJSQ). Insomnia was assessed using the Athens Insomnia Scale (AIS). Stepwise multiple logistic regression analyses were conducted. In a stepwise multivariate logistic regression analysis, it was found that the higher subdivided stress group by “role conflict” (OR = 5.29, 95% CI, 1.61–17.32) and anxious temperament score (OR = 1.33; 95% CI, 1.19–1.49) was associated with the presence of insomnia using an adjusted model, whereas other factors were excluded from the model. The study limitations were the sample size and the fact that only Japanese local government employees were surveyed. This study demonstrated the relationships between workers’ anxious temperament, role conflict, and insomnia. Recognizing one’s own anxious temperament would lead to self-insight, and the recognition of anxious temperament and reduction of role conflict by their supervisors or coworkers would reduce the prevalence of insomnia among workers in the workplace.

## Introduction

Insomnia impairs daytime functioning [1], reduces the quality of life [2], and contributes toward the economic burden of healthcare costs [3, 4] and losses in work performance (e.g., absenteeism and presenteeism) [5]. Non-depressed people with insomnia reportedly have a twofold risk of developing depression, compared to people with no sleep difficulties [6]. Insomnia is an independent indicator of suicide ideation even taking into account the core symptoms of depression such as depressed mood and anhedonia [7]. Employees’ insomnia can have significant effects on an organization’s performance, due to impairments in concentration, communication skills, decision-making, and flexible thinking [8]; therefore, a prevention strategy for insomnia is an urgent issue in the workplace. Some studies have demonstrated the relationship between insomnia and various types of occupational stress, such as job demand, job control, social support, job insecurity, organizational justice, intragroup conflict, job strain, effort–reward imbalance, employment level, and shift work [8–20].

Temperament has been defined as genetic personality factors that stay stable over time and establish an individual’s mood, reactivity, and energy at baseline. [21]. Temperament defines personality; and personality is argued to be developed through daily life experiences [22]. Akiskal formulated the modern concept of five affective temperaments and suggested that affective temperaments were the subclinical manifestations or phenotypes of mood disorders, representing one end of the continuum of affective illness, and subsequently developed the Temperament Evaluation of Memphis, Pisa, Paris, and San Diego-Auto (TEMPS-A) questionnaire for temperament research and clinical purposes [23–25]. A previous study found that unlike personality, temperaments assessed by the TEMPS-A did not change considerably over six years. [26]. Many studies have reported a relationship between temperaments and mental problems (e.g., suicide) [27, 28], mental status in non-clinical populations [29], depressive symptoms [30–32], mood disorders [33–37], anxiety disorders [38], alcohol abuse or dependence [39, 40], and substance abuse [41, 42]. In the workplace, a depressive temperament has been reported to be a kind of work-oriented personality [43], hyperthymic temperament has been reported to be a type of hyper-adapted personality [44]. A relationship between temperament and a subjective sleep pattern has been reported, and depressive, cyclothymic temperaments have been shown to be related to more dysfunctional sleep patterns; sleep-onset latency, the number of awakenings during the total sleep period, sleep quality, and hyperthymic temperament have shown an inverse and favorable sleep pattern [45].

We demonstrated the significant effects of temperaments on occupational stress in our previous study. For example, a hyperthymic temperament plays a protective role against one’s own job control, role ambiguity, job future ambiguity, an irritable temperament plays a vulnerable role against one’s own social support from supervisors, role conflict, variance in workload, intragroup conflict, and anxious temperament plays a vulnerable role against one’s own social support from coworkers, job future ambiguity [46]. Examining the effects of temperament on occupational stress was considered important to understand the relationship between insomnia and occupational stress among employees. However, temperaments have received little attention in occupational safety and health research. Moreover, previous studies on the relationship between occupational stress and insomnia did not evaluate the individual effects of temperaments, and therefore, this study seeks to extend and develop the findings of the previous study.

We hypothesized that individual temperament can play an important role in the relationship between occupational stress and insomnia. The aim of this study was to clarify the relationships between temperaments, occupational stress, and insomnia among local government employees.

## Materials and methods

### Subjects

We distributed self-administer, anonymous questionnaires to 172 Japanese day-shift workers in local government between August and September 2014. One hundred and thirty-three workers completed the questionnaire (response rate: 77.3%). All participants gave their verbal informed consent to participate as volunteers, and understood that there was no penalty for choosing not to participate.

### Ethics statement

The study design was approved by the Human Subjects Review Committee at Osaka City University (authorization number: 2969). All data were stored only in our database, and the employer did not have access to the data or know who had participated in the study.

### Demographic and work-related variables

Demographic variables included age and gender. Work-related variables included service years, position classification (non-managerial, managerial), occupation (clerical, professional), and type of employment (regular, temporary).

### Measures

#### Measures of temperament

Temperaments were assessed using the full version of the TEMPS-A, developed by Akiskal et al. [23, 25]. The reliability and validity of the Japanese version have been established [35]. The TEMPS-A assesses emotional, cognitive, psychomotor, interpersonal, and vegetative (such as sleep and sexual desire) dimensions that arguably play a vulnerable or adaptive role in an evolutionary context, and in the predisposition to major mood disorders [23]. The TEMPS-A is a true–false questionnaire that measures affective temperaments defining the bipolar spectrum. The instrument’s 110 items are divided into five types of temperaments, namely, depressive, cyclothymic, hyperthymic, irritable, and anxious. Higher scores suggest a greater magnitude of the temperaments.

A depressive temperament emerges as proneness to routine, self-blame, a shy nonassertive nature, sensitivity to criticism, self-denial, dependence, and preference to work for someone [23]. A cyclothymic temperament is characterized as being rather tempestuous, labile with rapid mood shifts, having variable sleep, energy, self-esteem, and socialization, being a dilettante and, perhaps by the same token, keen in perception and intense in emotions, and also a romantic [23]. A hyperthymic temperament has been associated with many positive traits: being upbeat, fun-loving, outgoing, jocular, optimistic, and confident; full of ideas and eloquent; active, a short-sleeper, but tireless; and having a preference for leadership; however, the temperament is also associated with single-mindedness, risk-taking, and a low likelihood of admitting one’s meddlesome nature [23]. An irritable temperament emerged with two characteristics—skeptical and critical—that might be considered intellectual virtues; otherwise, this temperament has the darkest nature of all: grouchy, complaining, dissatisfied, anger- and violence-prone, and sexually jealous [23]. An anxious temperament has been associated with many traits: worry, vigilance, tension, oversensitivity, unrestful sleep, and gastrointestinal symptoms [25, 47].

Two of the most commonly used assessments of temperament are the TEMPS-A and the Temperament and Character Inventory (TCI). The TCI was developed by Cloninger and colleagues [48]. The concurrent validity of the TEMPS-A with the TCI has been documented [25]. We used the TEMPS-A in this study because, with 110 items, it has the advantage of brevity over the TCI.

#### Measures of occupational stress

Occupational stress was assessed using the Generic Job Stress Questionnaire (GJSQ) developed by the National Institute for Occupational Safety and Health (NIOSH) [49]. The Japanese version of the GJSQ has demonstrated sufficient reliability and validity [50, 51]. We chose the GJSQ because it can assess multilateral aspects of occupational stress, including stress reactions, at the group and individual levels. The developers of the GJSQ permit use of its independent subscales to assess occupational stress [49]. We focused on the following five subscales (49 items) to assess occupational stress: quantitative workload, job control, role conflict, role ambiguity, and intragroup conflict, and we also chose two subscales measuring social support (by supervisors and coworkers, comprising eight items) that functions as a buffer factor, according to the results of previous studies [8–20]. The present study is based on the NIOSH job stress model [49]. For the job control and social support items, item descriptions are positively oriented, so that higher scores indicate lower stress. In contrast, all other items are negatively oriented, so that higher scores indicate greater stress.

A quantitative workload refers to the amount of work that a person has to deal with on a daily basis. Job control refers to the extent to which the individual feels that his or her tasks, workplace setting, and decisions at work are controllable. Role conflict measures how often workers experience role conflict with each other. Role ambiguity measures how clearly the worker understands what is expected of him or her for adequate task performance or assumption of a role. Intragroup conflict measures the harmony, conflict, or dialogue discrepancy in the group. Social support from supervisors and coworkers measures the existence of avenues for acquiring social support during work time.

#### Measures of insomnia

Insomnia was assessed using the Athens Insomnia Scale (AIS), which is a common global scale of insomnia and a validated instrument based on the International Classification of Disease, 10^th^ Revision (ICD-10) criteria [52, 53]. The Japanese version of the AIS has demonstrated sufficient reliability and validity [54]. The scale is a self-administered inventory consisting of eight items. The first five items assess difficulty in sleep initiation, awakening during the night, early morning awakening, total sleep duration, and the overall quality of sleep. The following remaining three items pertain to the consequences of insomnia on the next day: sense of well-being during the day, functioning during the day, and sleepiness during the day. Each item of the AIS was rated from 0 (no problem at all) to 3 (seriously problematic). The total score ranged from 0 to 24, and obtaining six or more points was defined as having insomnia. Participants were requested to choose rating items only if they had experienced sleep difficulty at least 3 times a week in the previous month. Based on previous studies, we defined the morbidity cut-off point on the AIS as 6 [54]. Individuals with AIS scores of more than 6 were categorized as the “Insomnia group” and displayed pathological insomnia, and those with AIS scores of less than 5 were categorized as the “Non-Insomnia group” and did not display any sleep problems.

### Statistical analyses

Univariate logistic regression analyses were performed to estimate the odds ratios (ORs) of demographic variables, work-related variables, five temperaments, and the seven GJSQ subscales with regard to the Insomnia group. The GJSQ subscales were subdivided into low, middle, or high categories according to the tertile scores. Subsequently, the ORs for belonging to the Insomnia group were estimated in the stepwise forward multivariate logistic analyses, and independent variables with p-value less than 0.20 were selected for the stepwise forward multivariate model. Differences were considered significant at p < 0.05. All statistical analyses were performed using SPSS version 21.0 software (SPSS Inc., Chicago, IL).

## Results

### Subjects’ characteristics

Table 1 shows the subjects’ characteristics, TEMPS-A temperament scores, GJSQ scores, and AIS scores. The total AIS scores were 4.5 ± 3.6. The “Insomnia group” consisted of 48 participants (36.1%; 16 male: 35.4% and 32 female: 36.4%) and displayed a mean AIS score of 8.5 ± 2.4. The “Non-Insomnia group” comprised 85 (63.9%) workers and exhibited a mean AIS score of 2.3 ± 1.6.

**Table 1:** Demographic variables, GJSQ scores, TEMPS-A scores, and AIS scores

		Range	n	(%)	Mean ± SD
Gender	Male		45	(33.8)	
	Female		88	(66.2)	
Position classification	Non-managerial		90	(67.7)	
	Managerial		43	(32.3)	
Occupation	Clerical		95	(71.4)	
	Professional		38	(28.6)	
Type of employment	Regular		101	(75.9)	
	Temporary		32	(24.1)	
Age					41.6 ± 11.9
Service years					14.0 ± 12.3
GJSQ scores	Quantitative workload	(11–55)			34.7 ± 7.4
	Job control	(16–80)			42.0 ± 11.9
	Role conflict	(8–56)			25.8 ± 8.2
	Role ambiguity	(6–42)			18.7 ± 5.2
	Intragroup conflict	(8–40)			19.3 ± 5.5
	Supervisors support	(4–20)			15.5 ± 3.3
	Coworkers support	(4–20)			16.1± 3.1
Temperaments	Depressive	(0–21)			8.1 ± 3.5
	Cyclothymic	(0–21)			4.4 ± 3.9
	Hyperthymic	(0–21)			5.3 ± 4.2
	Irritable	(0-Male 21, Female 22)			2.9 ± 3.2
	Anxious	(0–26)			6.3 ± 5.5
AIS scores	Total	(0–24)			4.5 ± 3.6
	Insomnia Group		48	(36.1)	8.5 ± 2.4
	Non-Insomnia Group		85	(63.9)	2.3 ± 1.6

GJSQ: Generic Job Stress Questionnaire

TEMPS-A: Temperament Evaluation of Memphis, Pisa, Paris, and San Diego-Auto questionnaire

AIS: Athens Insomnia Scale

### Correlations between temperaments and occupational stress

Table 2 shows Spearman’s correlation between the TEMPS-A temperament scores and occupational stress according to the GJSQ. The results were interpreted based on Guilford’s rule of thumb [55]. The depressive temperament score weakly correlated with job control. The cyclothymic temperament score moderately correlated with role conflict, role ambiguity, intragroup conflict, and weakly correlated with social support from coworkers. The hyperthymic temperament score moderately correlated with job control and role ambiguity. The irritable temperament score highly correlated with role conflict and intragroup conflict, moderately correlated with role ambiguity and social support from supervisors and from coworkers, and weakly correlated with the quantitative workload. The anxious temperament score moderately correlated with role conflict, social support from coworkers, and weakly correlated with social support from supervisors.

**Table 2:** Spearman's correlation between the TEMPS-A temperament scores and occupational stress

	1	2	3		4		5		6		7		8		9		10		11		12	
**1. qua**	-	-0.021	0.266	**	0.131		0.074		-0.11		-0.161		0.125		0.075		-0.006		0.217	*	0.109	
**2. jc**		-	0.041		-0.29	**	0.057		-0.045		0.003		-0.188	*	-0.094		0.234	**	-0.024		-0.119	
**3. rc**			-		0.306	***	0.461	***	-0.461	***	-0.455	***	0.124		0.266	**	0.012		0.433	***	0.27	**
**4. ra**					-		0.216	*	-0.219	*	-0.178	*	0.12		0.256	**	-0.29	**	0.226	**	0.137	
**5. int**							-		-0.408	***	-0.451	***	-0.003		0.238	**	0.114		0.326	***	0.126	
**6. sup**									-		0.652	***	-0.169		-0.168		0.006		-0.252	**	-0.203	*
**7. cow**											-		-0.143		-0.209	*	0.061		-0.287	**	-0.284	**
**8. dep**													-		0.412	***	-0.183	*	0.293	**	0.494	***
**9. cyc**															-		0.095		0.559	***	0.483	***
**10. hyp**																	-		0.173	*	-0.089	
**11. irr**																			-		0.468	***
**12. anx**																					-	

* p < 0.05,

** p < 0.01,

*** p < 0.001

TEMPS-A: Temperament Evaluation of Memphis, Pisa, Paris, and San Diego-Auto questionnaire

qua; quantitative workload, jc; job control, rc; role conflict, ra; role ambiguity, int; intragroup conflict, sup; support from supervisors, cow; support from coworkers, dep; depressive temperament, cyc; cyclothymic temperament, hyp; hyperthymic temperament, irr; irritable temperament, anx; anxious temperament

### Logistic regression analysis examining the association between temperaments, occupational stress, and insomnia

Table 3 shows the results of univariate logistic regression analysis using demographic variables (age and gender), work-related variables (service years, position classification, occupation, and type of employment), five temperaments, and each of the seven subdivided GJSQ subscales as independent variables, with the insomnia group as a dependent variable; ORs were calculated. It was found that the higher subdivided stress group by “role conflict” (OR = 8.60, 95% CI, 3.03–24.41), the lower subdivided stress group by “social support from coworkers” (OR = 3.00, 95% CI, 1.17–7.69), depressive (OR = 1.17, 95% CI, 1.05–1.31), cyclothymic (OR = 1.27, 95% CI, 1.14–1.42), irritable (OR = 1.26, 95% CI, 1.11–1.42), and anxious (OR = 1.36, 95% CI, 1.22–1.51) temperament score was associated with the presence of insomnia. Additionally, the middle subdivided stress group by “social support from supervisors” (OR = 2.71, 95% CI, 1.14–6.42) and by “social support from coworkers” (OR = 4.55, 95% CI, 1.76–11.75) was associated with the presence of insomnia.

**Table 3:** Analysis of risk factors for insomnia by crude and stepwise multiple logistic regression analysis.

		Crude model				Adjusted model†			
Temperaments and occupational stress		OR	(95% CI)	p		OR	(95% CI)	p	
Quantitative Workload	(Low)	1.00							
	Middle	1.01	0.40–2.59	0.98					
	High	1.99	0.83–4.77	0.12					
Job Control	(High)	1.00							
	Middle	1.41	0.55–3.67	0.48					
	Low	1.26	0.49–3.18	0.63					
Role Conflict	(Low)	1.00				1.00			
	Middle	2.63	0.90–7.69	0.08		2.12	0.63–7.14	0.224	
	High	8.60	3.03–24.41	<0.001	***	5.29	1.61–17.32	0.006	**
Role Ambiguity	(Low)	1.00							
	Middle	0.81	0.32–2.06	0.66					
	High	2.23	0.93–5.34	0.07					
Intragroup Conflict	(Low)	1.00							
	Middle	1.51	0.64–3.55	0.35					
	High	1.77	0.72–4.33	0.21					
Social Support from Supervisor	(High)	1.00							
	Middle	2.71	1.14–6.42	0.02	*				
	Low	0.72	0.29–1.79	0.48					
Social Support from Coworker	(High)	1.00							
	Middle	4.55	1.76–11.75	<0.01	**				
	Low	3.00	1.17–7.69	0.02	*				
									
Depressive		1.17	1.05–1.31	<0.01	**				
Cyclothymic		1.27	1.14–1.42	<0.001	***				
Hyperthymic		0.98	0.90–1.07	0.71					
Irritable		1.26	1.11–1.42	<0.001	***				
Anxious		1.36	1.22–1.51	<0.001	***	1.33	(1.19–1.49)	<0.001	***
									
Age		1.02	0.99–1.05	0.19					
Gender	(Female)	1.00							
	Male	0.97	0.46–2.04	0.93					
Position classification	(Non-managerial)	1.00							
	Managerial	1.67	0.79–3.51	0.18					
Occupation	(Clerical)	1.00							
	Professional	1.23	0.56–2.66	0.61					
Type of employment	(Regular)	1.00							
	Temporary	0.62	0.26–1.48	0.28					

OR: Odds Ratio. CI: Confidence Interval.

* p < 0.05,

** p < 0.01,

*** p < 0.001 compared with non-insomnia group.

Parenthesis denotes reference category.

†: Adjusted for all listed variables.

Additionally, Table 3 shows the result of stepwise multivariate logistic regression analysis using demographic variables (age and gender), work-related variables (service years, position classification, occupation, and type of employment), 5 temperaments, and the 7 subdivided GJSQ subscales as independent variables, with the insomnia group as a dependent variable; ORs were calculated. When entering these independent variables in a stepwise multivariate logistic regression analysis, it was found that the higher subdivided stress group by “role conflict” (OR = 5.29, 95% CI, 1.61–17.32), and anxious temperament score (OR = 1.33, 95% CI, 1.19–1.49) was associated with the presence of insomnia using an adjusted model, whereas other factors were excluded from the model.

## Discussion

This study identified the relationships between temperaments, occupational stress, and insomnia using the TEMPS-A, GJSQ, and the AIS among Japanese local government employees. The results of the present study indicate the relationships between anxious temperament, role conflict, and insomnia.

### Temperaments and insomnia

The anxious temperament is related to many psychological traits, such as, worry, awakening, tension, oversensitivity, unrestful sleep, and gastrointestinal symptoms [25, 47]. The anxious temperament has also been described to make strong predictions about anxieties [38] and suicide attempts [28]. It has been reported that worry, rumination, and a predisposition to worry increase vulnerability to sleep disorders [56]. Moreover, personality profiles associated with insomnia are characterized by the presence of neurotic depression, rumination, chronic anxiety, and a predisposition coupled with the process of conditioning (fear of sleeplessness–internalization of the fear–further emotional arousal and psychological activation). These factors have been found to contribute to the development of chronic insomnia [57]. Ottoni and colleagues reported that the depressive, cyclothymic, irritable, and anxious temperaments were related to worse sleep quality, and that the hyperthymic temperament was associated with better sleep quality [45]. The relationship between the anxious temperament and insomnia was found in this study, and the result is compatible with those of previous studies [45, 56, 57].

On the contrary, relationships between the depressive, cyclothymic, hyperthymic, and irritable temperaments and insomnia were not found in this study. The subjects in Ottoni’s study were recruited through an educational open-access website on bipolar disorder, which determined the inclusion of a significant number of subjects with psychiatric disorders and on psychotropic medication. Moreover, the authors have stated the generalizability of their results to the general population as a study limitation. Although there might be employees who work while having mental disorders, none of the subjects in this study were unemployed, and the results in the current study might therefore be more generalizable to the general population. Differences between the subjects might have had an impact on the results. In sum, workers’ anxious temperament might be associated with insomnia.

### Occupational stress and insomnia

The present study showed a relationship between role conflict and insomnia. Few studies have investigated the relationship between these variables. Knudsen and colleagues have reported that role conflict among full-time employees in the United States of America (USA) was positively associated with difficulties initiating sleep and non-restorative sleep, and that conflicting role demands might be related to organizational injustice and interpersonal stressors in the workplace [19]. Győrffy and colleagues have reported that intensive role conflict was associated with sleep disorders and that a higher workload might lead to more explicit role conflict among female physicians in Hungary [20]. On the other hand, no relationship between role conflict and insomnia was found among Japanese daytime workers in Nakata’s research [9]. Role conflict is reported to be a situation wherein employees face a lot of pressure from incompatible job demands like group interdependence, a different working style by subordinates, supervisors, and different requirements from people [58], and these factors might cumulatively lead to insomnia.

Some studies have demonstrated the relationships between insomnia and job demand, job control, social support, job insecurity, organizational justice, intragroup conflict, job strain, effort–reward imbalance, employment level, and shift work, among others [8–20]. No relationship was found with other types of occupational stress, except that between insomnia and role conflict in this study. The effects of the anxious temperament and role conflict on insomnia might be greater than those of other types of occupational stress. These differences might actually derive from differences in occupation, countries, or methods of evaluating occupational stress and insomnia.

### The prevalence rate of insomnia

The prevalence rate of insomnia was 36.1%, using the AIS for all study participants. This rate reportedly varies due to differences in applied definitions and diagnostic criteria [59]. Therefore, among studies using the AIS and demonstrating a relationship between insomnia and various types of occupational stress, the prevalence rate was from 23.2% to 39.2% [10–12, 14, 20]. Internet usage has become prevalent and various services are provided for 24 hours. As a result, the work life has changed dramatically, with the work–life balance becoming poor. In the modern society, the line between working hours and private time is ambiguous for 24 hours, workers’ physical and psychological stressors are increasing, and irregular circadian rhythms are a possibility. These remarkable changes in society might explain the increasing prevalence rate of insomnia, as shown by a comparison between the rate before 2003 [10, 14, 20] and that after 2007 [11, 12, 20].

### Prevention strategy for workers’ insomnia

First, workers’ recognition of their own personal temperaments, especially the anxious temperament, would contribute towards self-care and a decrease in the prevalence of insomnia. Second, it would be helpful for organizational staff to provide supportive guidance on stress-coping styles according to a cognitive-behavioral therapeutic approach that is based on each worker’s inclination towards an anxious temperament. Third, changing organizational policies, such as clarifying the role and content of diversified work, sharing information, and embracing diversity in the workplace would be needed to reduce role conflict. Fourth, supervisors and coworkers would have to receive training in accordance with the new policy. These strategies would reduce the prevalence of insomnia in the workplace.

### Limitations of this study

Several limitations in this study should be mentioned. First, the sample size was small, and only Japanese local government employees were surveyed. Second, occupational stress was evaluated through self-reports; thus, the results may have been influenced by response bias. Third, the moderate response rate for our survey questionnaire (77.3%) might have led to a selection bias. Fourth, it cannot be said with certainty from our data if the relationship between temperament, occupational stress, and insomnia is causal; nor can the direction of any such causality be established. For example, job stressors might affect scores on measures of temperament. This is unlikely; however, given the relative stability of temperament, as compared with stress. Finally, although the TEMPS-A scores may be related to mental status (e.g., depressive symptoms), mental conditions were not evaluated in this study. None of the subjects in this study were unemployed, but there might be employees who work while having mental disorders. Therefore, future studies would have to take sub-threshold mental disorders (e.g., depressive, anxiety symptoms) into consideration. In future research, a cohort or longitudinal design to address the relationships between temperament and the factors in the workplace would be beneficial.

## Conclusions

This study has demonstrated the relationships between workers’ anxious temperament, role conflict, and insomnia. Recognizing one’s own anxious temperament would lead to self-insight, and the recognition of the anxious temperament and a reduction of role conflict by supervisors or coworkers would reduce the prevalence of insomnia at workplace. In the future, we hope that temperaments will receive more focus within the study and practice of occupational safety and health.

## Supporting information

S1 Table GJSQ scores, Temperaments and AIS scores by gender (XLSX)

S2 Table GJSQ scores, Temperaments and AIS scores by type of employment (XLSX)

S3 Table GJSQ scores, Temperaments and AIS scores by position classification (XLSX)

S4 Table GJSQ scores, Temperaments and AIS scores by occupation (XLSX)

S5 Table Age (XLSX)

S6 Table Service years (XLSX)
